# Multi-Center Real-World Outcomes of Nivolumab Plus Ipilimumab and Chemotherapy in Patients with Metastatic Non-Small-Cell Lung Cancer

**DOI:** 10.3390/biomedicines11092438

**Published:** 2023-08-31

**Authors:** Walid Shalata, Alexander Yakobson, Yulia Dudnik, Forat Swaid, Mohammad Sheikh Ahmad, Ashraf Abu Jama, Ahron Yehonatan Cohen, Abed Agbarya

**Affiliations:** 1 The Legacy Heritage Cancer Center & Dr. Larry Norton Institute, Soroka Medical Center, Beer Sheva 84105, Israel; 2 Faculty of Health Sciences, Ben-Gurion University of the Negev, Beer Sheva 84105, Israel; 3 Department of Surgery, Bnai Zion Medical Center, Haifa 31048, Israel; 4 The Ruth and Bruce Rappaport Faculty of Medicine, Technion, Haifa 31048, Israel; 5 Institute of Endocrinology, Bnai Zion Medical Center, Haifa 31048, Israel; 6 Oncology Department, Bnai Zion Medical Center, Haifa 31048, Israel

**Keywords:** chemotherapy, ipilimumab, nivolumab, metastasis, non-small-cell lung cancer (NSCLC), progression free survival, overall survival, PD-1 inhibitor, PDL-1 inhibitor

## Abstract

Immune checkpoint inhibitors have become the standard of care in the treatment of metastatic non-small-cell lung cancer (NSCLC). The combination of nivolumab plus ipilimumab and chemotherapy has been shown to improve outcomes in terms of overall survival (OS) and progression-free survival (PFS). The aim of this study was to evaluate the outcomes of metastatic NSCLC treated in routine practice on the treatment regimen of the CheckMate 9LA protocol. Medical records of 58 patients treated at Soroka and Bnai Zion Medical Centers between May 2020 and February 2022 were analyzed. All patients were treated with a regimen of platinum-based chemotherapy combined with immunotherapy of nivolumab every three weeks and ipilimumab every 6 weeks. The patients received 2–3 cycles of chemotherapy according to the physician’s choice: platinum-based cisplatin or carboplatin with either pemetrexed or paclitaxel. The median PFS was 10.2 months, longer than that of the 9LA trial (6.7 months). Adenocarcinoma patients exhibited a higher median OS of 13.7 (range 5–33) months than squamous cell carcinoma (SCC) patients at 12.3 (5–20) months and PFS of 10.3 (4–33) months, while squamous cell carcinoma patients had a PFS of 9.2 (4–18) months. Patients whose programmed death ligand-1 (PD-L1) tumor expression level was ≥1% showed a higher median OS than those with PD-L1 expression of less than 1%. Treatment-related adverse events (TRAEs) were reported in 93.1% of patients, mostly grade 1 in severity. The first-line treatment of metastatic NSCLC patients in combination with nivolumab plus ipilimumab and chemotherapy can be given safely in routine clinical practice, with results comparable to those achieved in clinical trials of the regimen.

## 1. Introduction

Lung cancer is one of the most commonly diagnosed cancers worldwide and the most common cause of cancer-related death. Globally, there are an estimated 1.8 million new cases of lung cancer diagnosed every year [1,2,3].

In recent years, the 5-year survival rate of lung cancer in the United States has approached 20%. The prognosis of this disease is dependent on the stage at which the diagnosis is made [4]. The majority of lung cancer cases are diagnosed when the cancer has already metastasized beyond the lungs [5]. Over two-thirds of the patients diagnosed with lung cancer are over the age of 70 years old, whereas only 3% of patients are diagnosed under the age of 45 years old [1,3]. Non-small-cell lung cancer (NSCLC) accounts for approximately 80–85% of all lung cancer cases. Histologically, NSCLC is classified into several subtypes, including lung adenocarcinoma (LUAD), squamous cell carcinoma (SCC), large cell carcinoma, and adenosquamous carcinoma. [1,5,6]. The poor prognosis of NSCLC can be attributed to the initial diagnosis of 70% of patients when they are already at advanced stages (III-IV). As a consequence, NSCLC is associated with high morbidity and mortality rates [6,7].

The recommended treatment for unresectable stage III advanced NSCLC is a combination of chemotherapy and radiation therapy [8]. Platinum-based combinations have been the standard protocol and the primary option for treatment in such cases [7,8,9]. Metastatic NSCLC is associated with poor outcomes, typically resulting in a median survival of approximately 1 year. [7]. In the current decade, immune checkpoint inhibitors (ICI) have been introduced and approved for the treatment of NSCLC in the neo-adjuvant, adjuvant, locally advanced, and metastatic settings. ICI therapy works by blocking the interaction between programmed death ligand 1 (PD-L1) on tumor cells and programmed cell death protein 1 (PD-1) on T cells, thereby enabling T cells to effectively eliminate tumor cells. Recent studies have demonstrated that ICI monotherapy, as well as its combination with chemotherapy, improves survival outcomes and exhibits lower toxicity compared to chemotherapy alone in patients with advanced NSCLC [2,5,10,11,12]. The current and most promising ICIs are pembrolizumab and nivolumab plus ipilimumab. Pembrolizumab and nivolumab are both humanized IgG4 monoclonal antibodies that specifically target PD-1, while ipilimumab is a fully humanized IgG1 antibody that targets cytotoxic T-lymphocyte-associated protein 4 (CTLA-4) [10,11,13]. Nivolumab, in combination with ipilimumab, has been tested as a treatment option in comparison to chemotherapy alone in patients with metastatic NSCLC. Additionally, the combination of nivolumab and ipilimumab has been studied in combination with chemotherapy, compared to chemotherapy alone, for the treatment of metastatic NSCLC [14,15].

The binding of PD-1 to its ligands, PD-L1 or PD-L2, inhibits the cytotoxic T-cell response, thereby blocking this immune pathway [16,17]. Nivolumab promotes tumor recognition by activating T cells, and ipilimumab stimulates the immune system [10]. According to the CheckMate 9LA trial report, the combination of nivolumab plus ipilimumab with chemotherapy demonstrated longer progression-free survival (PFS) and overall survival (OS) compared to traditional platinum-based therapy in patients with NSCLC, regardless of their PD-L1 expression levels [14]. In the CheckMate 227 trial, it was observed that in patients with NSCLC who had PD-L1 expression levels equal to or greater than 1%, the combination of nivolumab plus ipilimumab resulted in longer OS compared to traditional platinum-based therapy. Moreover, in patients with PD-L1 expression levels equal to or greater than 50%, the combination therapy demonstrated prolonged PFS as well as the OS when compared to platinum-based therapy [15].

In order to contribute to the existing but limited pool of data on real-world outcomes, this study presents a report on 58 patients diagnosed with NSCLC who underwent treatment based on the CheckMate 9LA trial, including nivolumab plus ipilimumab and chemotherapy. The study focuses on evaluating PFS, OS, and treatment-related adverse events (TRAEs). By providing these additional data, we aim to further enhance our clinical understanding of the effectiveness and safety of this treatment approach in real-world clinical settings.

## 2. Materials and Methods

### 2.1. Study Population

This study was conducted as a multi-institutional, retrospective cohort study. Patient recruitment was carried out through electronic medical records, and the study included all NSCLC patients who received nivolumab plus ipilimumab therapy in combination with chemotherapy between May 2020 and February 2022. The last date of follow-up was on 1 March 2023. Patient data in this study was collected and analyzed for various parameters, including the treatment regimen administered, the start and end dates of therapy, the date of the last follow-up, the duration of OS and PFS, response rate (RR), disease control rate (DCR), PD-L1 expression levels, additional mutations, and TRAEs. These parameters were assessed to provide comprehensive information about the treatment outcomes, biomarkers, and safety profile associated with the nivolumab plus ipilimumab therapy in combination with chemotherapy for NSCLC patients. The study was approved by the Institutional Review Boards of Soroka Medical Center (approval no. 0316; on 2 December 2021) and Bnai Zion Medical Center (approval no. 0023-22-BNZ).

Each study patient’s case was presented and discussed by a multidisciplinary medical team. This team consisted of various specialists, including a general medical oncologist, a radiation oncologist, an imaging physician, a nuclear physician, a pulmonologist, a pathologist, and a thoracic surgeon. The discussions held by these teams were based on the patient’s medical condition, pathology reports, and imaging records.

Each patient was assigned a primary physician who assumed responsibility for overseeing their treatment course. For patients with previous advanced or metastatic diagnoses, the primary treatment providers were typically medical oncologists. The treatment plans for these patients were generally based on the recommendations provided by the National Comprehensive Cancer Network (NCCN), a recognized authority in cancer care and treatment guidelines. The multidisciplinary approach and adherence to established recommendations aimed to ensure comprehensive and evidence-based care for the patients in the study [18].

### 2.2. Inclusion Criteria

Age: 18 years or older.

Lung Cancer Diagnosis: Histologically confirmed stage IV or recurrent non-small-cell lung cancer (NSCLC).

Treatment: Patients received a combination of chemotherapy and nivolumab plus ipilimumab as first-line systemic therapy for NSCLC.

Performance Status: Eastern Cooperative Oncology Group (ECOG) performance status scores ranging from 0 to 4.

Additional inclusion criteria indicated patients were also eligible if they received their first cycle of treatment up to one year before the study period.

In addition to the aforementioned inclusion criteria, it was required that the patients had not been administered any prior systemic therapy for their advanced or metastatic disease. The patients included in the study were treated specifically at the Soroka and Bnai Zion Medical Centers or had a complete follow-up history documented in the medical records of these medical centers.

### 2.3. Exclusion Criteria

Treatment with chemotherapy only, immunotherapy only, or chemotherapy plus IO other than ipilimumab and nivolumab (such as cemiplimab or pembrolizumab).

Follow-up of less than 1 year.

Tumor mutations detected for EGFR, ALK, ROS, MET, and BRAF.

Patients with two malignant primaries, except basal cell carcinoma or SCC of skin.

Previous ipilimumab and nivolumab treatment for malignant tumor.

Patients with a history of acute cardiovascular or cerebrovascular accidents less than 1 year before administration of treatment for lung cancer.

Sixty-five patients were screened, of which fifty-eight met the eligibility criteria. Detailed information regarding these patients can be found in Table 1 and Table 2 of the study.

### 2.4. Treatment Administered

#### 2.4.1. NSCLC Patients Diagnosed with Adenocarcinoma, Pleomorphic, and Adenocarcinoma with Neuroendocrine Features (*n* = 44)

In this cohort, the patients received two cycles of chemotherapy as determined by the treating physician. On the first day of the treatment cycle (cycle duration of 3 weeks), the following drugs were administered intravenously: cisplatin 75 mg per square meter of body-surface area or carboplatin at a dose calculated based on the area under the concentration-time curve (AUC) (6.5 or 4 mg per milliliter per minute, depending on the patient’s performance status), in combination with pemetrexed 500 mg per square meter of body-surface area. In addition, nivolumab was administered at a dose of 360 mg IV (intravenously) every 3 weeks, and ipilimumab was given at a dose of 1 mg/kg IV every 6 weeks. After the initial two cycles of therapy, the platinum drug was omitted, and pemetrexed, nivolumab, and ipilimumab were continued as maintenance therapy.

This treatment protocol was continued for up to 2 years or until there was unacceptable toxicity or disease progression. All patients received premedication for pemetrexed as per institutional guidelines with folic acid, vitamin B12, and glucocorticoids.

#### 2.4.2. NSCLC Patients Diagnosed with Squamous Cell or Adenosquamous Carcinomas (*n* = 14)

In this cohort, the treatment regimen included carboplatin, administered at a dose calculated to achieve an AUC of 6.5 or 4 mg per milliliter per minute, depending on the patient’s performance status, along with paclitaxel 175 mg per square meter of body-surface area. Additionally, patients received nivolumab 360 mg IV every 3 weeks, and ipilimumab 1 mg/kg IV every 6 weeks. Following the initial 2 cycles, the treatment protocol continued with nivolumab at a dosage of 360 mg every 3 weeks and ipilimumab at a dosage of 1 mg/kg every 6 weeks. This maintenance phase was maintained for up to 2 years or until there was unacceptable toxicity or disease progression.

### 2.5. Data Analysis

Descriptive statistics were employed to summarize the treatment outcome measures, including OS, PFS, and adverse events (AEs). Median (m), range, frequencies (*n*), and percentages were calculated using the EXCEL program.

OS was defined as the duration of time from the initiation of first-line treatment to the occurrence of death, irrespective of the cause. PFS was defined as the duration of time from the initiation of first-line treatment to either clinical or radiological progression of the disease or death from any cause. The evaluation of disease progression was conducted according to the Response Evaluation Criteria In Solid Tumors (RECIST) criteria version 1.1. Additionally, the safety profile of the study treatment was evaluated by analyzing dose-limiting toxicities and treatment-related adverse events (TRAEs).

## 3. Results

### 3.1. OS and PFS

Follow-up time was 34 months (from May 2020 until March 2023) for all patients (since the first patient started treatment).

Median OS (mOS) for all patients was 13.37 months (range 5–33), and the median PFS (mPFS) was 10.22 months (range 4–33).

### 3.2. OS and PFS in Comparison between Various Quantitative Samples (Table 2)

The group of patients diagnosed with LUAD was compared to the group of SCC patients. The LUAD group had better PFS outcomes than the SCC: the median PFS was 10.3 months (range 4–33) for LUAD vs. 9.2 months (range 4–18) for the SCC group. For the LUAD group, the median OS was 13.69 months (5–33). For patients with SCC, the median OS was 12.3 months (5–18).

Statistical analysis for patients with other NSCLC types was not carried out because of the small sample size.

PFS and OS for various subgroups of patients according to cancer type, PD-L1 expression, mutation profile, and ECOG status are presented in Table 1 and Table 2. Comparing the non-squamous patient and squamous patient subgroups, it was noted that the mOS and mPFS were longer in the non-squamous patients than in all other categories. The mPFS and mOS of pleomorphic carcinoma patients and LUAD patients with neuroendocrine features were 12 months each, compared with the mPFS and mOS for adenosquamous of 17 months (all patients are still under treatment).

The highly (equal to or over 1%) PD-L1 levels revealed better results than lower levels of PD-L1 (less than 1%) regarding mOS and mPFS for the non-squamous patient compared with the squamous patient.

Patients with ECOG 0 and 1 had higher mOS.

### 3.3. Treatment-Related Adverse Events

TRAEs were reported by 54 patients (93.1%) (Table 3). No patient experienced grade 3 or 4 TRAEs. A single patient died due to TRAE sepsis (grade 5). No patient had to stop treatment due to TRAEs. Most common TRAEs, anemia, rash, fatigue, and diarrhea, were reported as Grade 1.

### 3.4. Overall Response Rate

In this cohort, all patients received a combination of nivolumab plus ipilimumab in addition to chemotherapy (patients received a platinum-based agent in combination with other cytotoxic drugs such as paclitaxel and pemetrexed, the particular combination depending on the lung cancer histologic type or physician choice). Most patients (88%) received two cycles of chemotherapy with the ICIs, nivolumab plus ipilimumab; 12% of the patients were administered three cycles. The overall response rate (ORR) one year after initiation of treatment was evaluated as complete response, partial response, or stable disease in 70.6% of patients (Table 2). Disease progression (or disease relapse) was noted in 17 patients with a follow-up of at least 1 year, among whom six patients (10.3%) had disease progression in the brain, five (8.6%) in the liver, two (3.44%) in liver and brain in the same period, two (3.44%) in the lung, one (1.7%) in the adrenal, and one patient (1.7%) in both bone and brain in the same period.

## 4. Discussion

Over the past few years, with the introduction of IO in standard treatment protocols, important progress has been achieved in the treatment of advanced NSCLC, including reaching a 5-year survival of 18–33% [19].

Choosing the optimal treatment is still challenging, taking into account there is a wide range of treatment options available and the lack of direct comparison between them. Today, most patients are presented to multidisciplinary teams, and all treatment options are considered. The treatment decision is made based on the patient’s clinical features, such as age, performance status (PS), extent/volume of the disease, symptoms, and pathologic and molecular biomarkers.

The CheckMate 9LA clinical trial regimen of nivolumab 360 mg every 3 weeks plus ipilimumab 1 mg/kg every 6 weeks with only two cycles of platinum doublet-based chemotherapy has shown long-term durable efficacy and a manageable safety profile, leading to the implementation of the regimen in the first-line treatment setting for patients with metastatic NSCLC regardless of PD-L1 status [12]. In our cohort of 58 patients, the RR, including complete response (CR), partial response (PR), stable disease (SD), and progressive disease (PD), differed from that reported in the CheckMate 9LA trial, where the CR rate was 2%, compared to 6.89% in our cohort; the PR rate was 36%, whereas in our cohort, it was higher at 44.82%, and the SD rate observed in the 9LA trial stood at 45%, contrasting with our cohort’s rate of 19%. Lastly, the PD rate in the 9LA trial was 9%, while in our cohort, it was 29.3%. In CheckMate 9LA, mOS was 15.6 months (95% CI 13.9–20.0) for the chemo-immunotherapy cohort, and mOS was 10.9 months (95% CI 9.5–12.6) for the chemotherapy-only cohort. The present study demonstrated a mOS of 13.37 months for the chemo-immunotherapy cohort, which was longer than the mOS of the chemotherapy-only cohort of the CheckMate 9LA study; however, it was shorter than the mOS of the chemo-immunotherapy 9LA cohort.

In the subgroup analysis, the mOS exhibited variations among different patient groups. Specifically, patients diagnosed with LUAD displayed more favorable outcomes, with a mOS of 13.69 months (5–33), in contrast to the 9LA trial, where the mOS was 17 months (14–20). For patients with SCC, the mOS was 12.3 months (5–20), as opposed to the 9LA trial results of 14.5 months (13.1–19.4). In cases of pleomorphic carcinoma and LUAD with neuroendocrine features, the mOS reached 12 months, and these patients are still undergoing treatment. Similarly, for adenosquamous carcinoma, mOS has reached 17 months, with all patients still undergoing treatment.

In addition, the 9LA study showed mPFS of 6.7 months (95% CI 5.6–7.8) for the chemo-immunotherapy cohort and mPFS of 5.0 months (95% CI 4.3–5.6) for the chemotherapy-only cohort. As noted, the current study exhibited an mPFS of 10.22 months for chemo-immunotherapy, which is 1.5 times longer than the chemo-immunotherapy cohort and the PFS chemotherapy-only cohort of the original 9LA study [13,14]. The rationale of this four-drug combination is that while both nivolumab and ipilimumab have non-overlapping anti-cancer mechanisms, chemotherapy delivered during the induction phase may increase tumor antigen and reduce inhibitory signaling, leading to activation of the host immune system. Accordingly, by using this particular combination, the two cycles of chemotherapy would support initial disease control and regression. The IO treatment would result in a long duration of efficacy. This combination seems to be both physician- and patient-“friendly” due to the use of only two cycles of chemotherapy, which can have a positive impact on the patient’s QoL without reducing the treatment efficacy.

The present investigation reports on the experience of Soroka and Bnai Zion Medical Centers with the CheckMate 9LA protocol, based on 58 patients. There are some differences between our experience and the reported data in the CheckMate 9LA protocol. In terms of patients’ characteristics, in our study, most of the patients were males, as expected, but in contrast to the 9LA population, 40% of them were with ECOG-2, 25% were very symptomatic, all of them were active or past smokers, 63% had at least one comorbidity, and 65.5% had negative PD-L1 in their tumor tissue.

Due to the long turnaround for molecular testing, seven (12%) patients received three cycles of chemotherapy, and the IO combination was administered starting from the second cycle. With a median follow-up of 12.2 months, the mPFS and mOS for the entire group were 10.22 months and 13.37 months, respectively, with better outcomes for adenocarcinoma patients as expected (19).

In terms of PD-L1 expression, the results were different between mPFS and mOS. While the best mPFS was for the group of PD-L1 < 1%. The best mOS was obtained in the group of patients expressing ≥1% PD-L1. (The cohort of patients expressing PD-L1 equal or over 50% was small (*n* = 4) and not sufficient for statistical analysis).

The response rate for this study regimen was more than expected, with 70% DCR, including 64% partial and complete response [14,15]. The percentage of TRAEs reported in our cohort was less than in clinical trials [14,15]. The reported toxicity of any grade was 93.1% (mostly grade 1, no grade 3–4, and without treatment discontinuation or dose interruption, with one death due to sepsis (grade 5)).

These differences in mPFS and mOS, in our experience compared to the CheckMate 9LA trial [14], could be explained by taking into account the limitation of this real-world retrospective data collection and the small sample size of patients included. The patient characteristics were different from the highly selected population in the 9LA trial. Our patients were more symptomatic, had poorer ECOG status, and had poor prognostic disease characteristics. This may explain the mOS of 2.5 months less than what was expected despite the relatively high mPFS, which was higher than was expected compared to the original CheckMate 9LA trial [14]. The explanation for this higher mPFS may be related to less stringent radiologic evaluation for treatments in the real-world clinical setting, especially in the era of IO, where RECISTs are not routinely used and, in many cases, have been replaced by the use of immune response evaluation criteria in solid tumors (iRECIST) [20]. The iRECIST is not a well-established method for routine clinical use in many medical centers, and in many cases, there is an overestimation of “atypical responses” like pseudoprogression or hyperprogression [21,22]. In addition, it is difficult to assess the mPFS in real-world retrospective trials because of intra-observer variation in radiologic response evaluation, especially once taking into account the clinical status of a patient.

A pragmatic efficacy endpoint that may be used in real-world evidence (RWE) trials, where radiologic evaluation is less structured and standardized, is “time-to-treatment discontinuation” (TTD) [23]. TTD is defined as the time from treatment starting day to the day of treatment discontinuation or death and is associated with PFS across different systemic therapeutic modalities. For this reason, the higher mPFS calculated here may reflect the real time of treatment, more so than a real mPFS, taking into account the treating physician’s intention to maximize the time using this combination and the unstructured radiologic evaluation. The crucial question would be whether the physician’s judgment about continuing treatment will negatively impact the patient response and efficacy for the next treatment line.

Treatment beyond progression is well defined in the therapy of NSCLC with specific agents such as tyrosine kinase inhibitors in special situations [24,25], but it is still not completely understood in treatment with IO [26]. The resistance mechanisms for this particular nivolumab plus ipilimumab combination are still not understood and may involve different pathways for IO, such as tumor microenvironment (TME) [27], as well as for chemotherapy, such as adaptive resistance mechanisms [28]. Taking these complex resistance mechanisms into account is important for routine clinical management in order to identify disease progression in a timely fashion and to switch to the next available treatment line before the cancer cells progress and become more resistant.

TRAEs were less severe in our study compared to the CheckMate 9LA trial (9LA) in terms of grades and the necessity to discontinue treatment. This finding is probably due to the clinical experience of the treatment team and the awareness of the need for close monitoring that has been gained from the increasing use of immunotherapy and chemo-immunotherapy in clinical practice. Diagnosing these adverse events in a timely fashion and treating them appropriately benefited the patients and probably improved outcomes.

Our study’s limitations include its reliance on data from only two institutions. To validate these findings, future research should encompass data from multiple institutions or countries and larger patient cohorts. Additionally, extending the follow-up period, as with the original study, is necessary to thoroughly evaluate the various endpoints. Finally, the accuracy of the performance status designation may be in question. In a real-world setting, when a treating physician’s intention is to maximize the first-line treatment time using IO and chemotherapy combination, ECOG status may be less strictly monitored, and therefore, the status could have been better than ECOG 2 and inaccurately recorded ECOG 2. Our study shows the importance of detailed recording of patient information, including ECOG scores and other data, as it may prove crucial in future research and studies. Accurate and comprehensive documentation can significantly contribute to the advancement of medical knowledge and ultimately benefit patient care.

## 5. Conclusions

The 9LA combination is a new and unique treatment option for patients with mNSCLC.

Here, we report the first real-world experience with this combination, and our results have shown similar results of efficacy and tolerability and support the use of this combination in this setting.

By combining minimal chemotherapy (only two cycles of platinum-based) with two different immunotherapies (PD1 inhibitor and CTLA-4), the efficacy was comparable to other available combinations, including those with full doses of chemotherapy. The efficacy was obtained regardless of the PDL-1 status and histology, especially in specific patient populations with high unmet needs such as PDL-1 negative, squamous histology, and patients with brain metastasis.

This combination offers a tolerable profile of toxicity as opposed to other chemotherapy-containing regimens. Based on this, good efficacy, and tolerable profile of toxicity, this first-line treatment of metastatic NSCLC patients in combination with nivolumab plus ipilimumab and chemotherapy can be given safely in routine clinical practice.

## Figures and Tables

**Table 1:** Baseline disease characteristics of the study population (*n* = 58).

Characteristic		Median (Range)
Age (years)		68.6 (40–86)
	Male	70.1 (52–86)
	Female	63.8 (40–81)
Gender		Frequencies (percentage)
	Male	44 (75.8)
	Female	14 (24.2)
Smoking status (at diagnosis)		
	Current	44 (75.8)
	Former	14 (24.2)
	Never	0 (0)
Underlying medical condition		
	Chronic obstructive pulmonary disease (COPD)	9 (15.5)
	Cardiac events	13 (22.4)
	Hyperlipidemia	19 (32.75)
	Diabetes type 2	12 (20.6)
	Hypertension	18 (31)
	Cerebrovascular accident (CVA)	4 (6.8)
ECOG status		
	0	10 (17.2)
	1	25 (43.1)
	2	23 (39.6)
Histology		
	Adenocarcinoma	42 (72.4)
	Squamous cell carcinoma	13 (22.4)
	Others *	3 (5.16)
Co-mutations		
	STK11	7 (12)
	TP53	14 (24.1)
	KRAS	17 (29.42)
PD-L1 status	PD-L1 expression ≥ 1%	PDL1 expression < 1%
Non-SCC **	17 (29.5)	27 (46.4)
SCC	3 (5.1)	11 (19)
Co-mutations		
14 TP53 (24.1)	17 KRAS (29.42)	7 STK11 (12)

Abbreviation: ECOG, Eastern Cooperative Oncology Group; PD-L1, programmed cell death ligand 1; NSCLC, non-small-cell lung cancer; SCC, squamous cell carcinoma; KRAS, Kirsten Rat Sarcoma Virus; TP 53, tumor protein p53; STK11, Serine/Threonine Kinase 11. Note: Immunohistochemistry was performed on tissue biopsy samples. The tumor’s PD-L1 expression was measured using Ventana’s XT Benchmark using IHC PharmDx (clone 22C3, Dako) UltraView detection kit (FDA approved, Ventana). * Adenosquamous, adenocarcinoma with neuroendocrine features, and pleomorphic carcinoma. ** The Non-SCC group consisted of 42 patients with adenocarcinoma, 1 patient with pleomorphic, and 1 patient with adenocarcinoma with neuroendocrine features.

**Table 2:** Results of response rate *, overall survival, and progression-free survival of NSCLC patients to nivolumab plus ipilimumab and chemotherapy **.

Characteristic Cycles of Chemotherapy Administered		Frequencies (Percentage)	
	2	51 (88)	
	3	7 (12)	
		**Median (range)**	
**Time from diagnosis to availability of molecular test results (weeks)**		3.4 (2–5)	
**Type of response**		**Frequencies (percentage)**	
	Complete response	4 (6.89%)	
	Partial response	26 (44.82%)	
	Stable disease	11 (19%)	
	Progressive disease	17 (29.3%)	
		**mOS months (range)**	**mPFS** **months (range)**
**Study population**	(*n* = 58)	13.37	10.22
**NSCLC type**	Non-SCC (*n* = 44)	13.75 (6–33)	10.3 (4–33)
	SCC (*n* = 14)	12.3 (5–18)	9.2 (4–18)
**ECOG status**			
	0 (*n* = 11)	15 (8–23)	10.4 (7–18)
	1 (*n* = 26)	13.88 (6–32)	11.52 (6–28)
	2 (*n* = 21)	12.75 (4–33)	8.73 (5–33)
**PD-L1 expression**			
Non-SCC ***			
	PD-L1 < 1%	13.39 (5–33)	10.89 (4–33)
	PDL1 ≥ 1%	14.58 (8–29)	9.88 (5–24)
SCC			
	PD-L1 < 1%	13 (6–20)	10.4 (4–18)
	PDL1 ≥ 1%	10.1 (6–24)	8.3 (4–12)

Note: * Data presented as months (range), maximum follow-up at first year of treatment. ** Data presented as months (range), maximum follow-up: 33 months. Abbreviations: m denotes median; OS, overall survival; PFS, progression-free survival; SCC, squamous cell carcinoma; NSCLC, non-small-cell lung cancer; ECOG, Eastern Cooperative Oncology Group; *** The non-SCC group was composed of 42 patients with adenocarcinoma, 1 patient with pleomorphic, and 1 patient with adenocarcinoma with neuroendocrine features.

**Table 3:** Treatment-related adverse events of nivolumab plus ipilimumab and chemotherapy administered to NSCLC patients.

Type of Adverse Events	All Grades *n* (%)	Grade 1 *n* (%)	Grade 2 *n* (%)
Anemia	54 (93.1%)	49 (84.5%)	5 (8.6%)
Skin	25 (43.1%)	23 (39.7%)	2 (3.44%)
Fatigue	14 (24.1%)	12 (20.6%)	2 (3.44%)
Diarrhea	11 (18.9%)	9 (15.5%)	2 (3.44%)
Transaminitis	6 (10.3%)	5 (8.6%)	1 (1.7%)
Thyroiditis	6 (10.3%)	5 (8.6%)	1 (1.7%)
Vomiting	4 (6.9%)	4 (6.9%)	
Thrombocytopenia	3 (5.17%)	2 (3.44%)	1 (1.7%)
Nausea	2 (3.44%)	2 (3.44%)	
Arthralgia	2 (3.44%)	2 (3.44%)	
Neutropenia	2 (3.44%)	1 (1.7%)	1 (1.7%)
Hepatitis	1 (1.7%)	1 (1.7%)	
Death (grade 5)	1 (1.7%)		

Note: % denotes percentage of total (*n* = 58) study participants.

## Data Availability

The data either reside within the article itself or can be obtained from the authors upon making a reasonable request.
